# Chest wall granuloma associated with BCG vaccination presenting as hot abscess in an immunocompetent infant

**DOI:** 10.1186/s13019-015-0231-z

**Published:** 2015-03-04

**Authors:** Hyun Seung Lee, Kyung Jin Seo, Jae Jun Kim

**Affiliations:** 1 Department of Pediatrics, Uijeongbu St. Mary’s Hospital, The Catholic University of Korea College of Medicine, Uijeongbu, South Korea; 2 Pathology, Uijeongbu St. Mary’s Hospital, The Catholic University of Korea College of Medicine, Uijeongbu, South Korea; 3 Department of Thoracic and Cardiovascular Surgery, Uijeongbu St. Mary’s Hospital, The Catholic University of Korea College of Medicine, 271 Cheonbo Street, Uijeongbu City, Gyeonggi-do 480-717 Korea

**Keywords:** BCG vaccination complication, Hot abscess, Chest wall abscess

## Abstract

Bacillus-Calmette-Gue´rin (BCG) vaccine is a live attenuated vaccine to prevent tuberculosis by cell mediated immune response and is routinely administered early after birth. Although it is considered to be a very safe vaccine, sometimes a variety of complications may develop. Herein we describe a clinically unusual case of chest wall granuloma considered to be induced by BCG, presenting as hot abscess, and developed 7 months after BCG vaccination in an immunocompetent infant. The diagnosis was made based on the history, histopathology and virological studies. We suggest, although very rare, a BCG disease should be considered as a differential diagnosis in case of chest wall abscess, even if this is presenting as a hot abscess and even in immunocompetent infants if their age is related to BCG vaccination complications.

## Background

Bacillus-Calmette-Gue´rin (BCG) vaccine is a live attenuated vaccine to prevent tuberculosis by cell mediated immune response and is routinely administered early after birth. Although it is considered to be a very safe vaccine, sometimes various complications may develop. Complications induced by BCG vaccine are very rare, estimated to be 0.01-3.6% [1]. The most common complications induced by BCG vaccination are regional lymphadenitis and local subcutaneous abscess around the administration site. Herein, we report a clinically unusual case of chest wall granuloma considered to be induced by BCG, presenting as hot abscess, and developed 7 months after BCG vaccination in an immunocompetent infant.

## Case presentation

**Figure 1:**
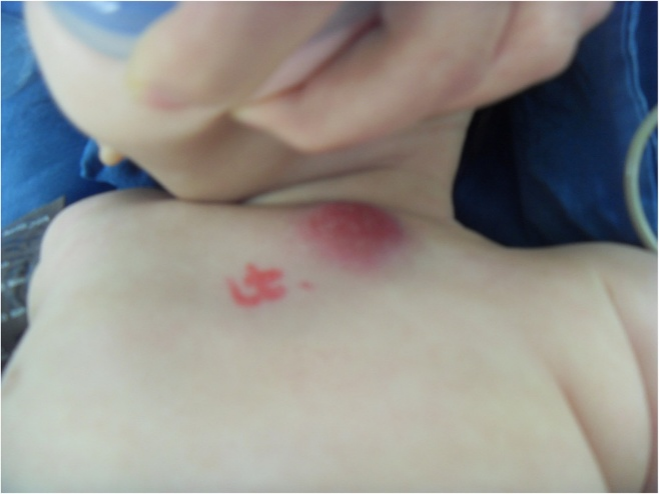
**The patient has a tender erythematous immobile firm mass along the left second intercostal space of the midclavicular line, measuring about 3 x 2 cm in size.**

**Figure 2:**
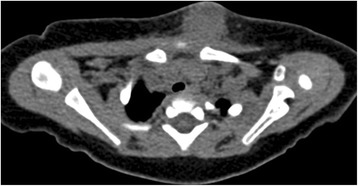
**The chest CT shows a 2 x 3 cm ill-defined soft tissue density lesion with abscess formation, adjacent infiltration into subcutaneous fat and the pectoralis major muscle.** Also there is no lesion in the pulmonary parenchyma.

**Figure 3:**
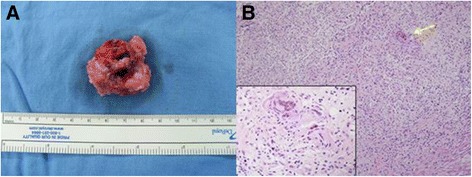
**Chest wall abscess histopathologic findings (A: gross B: microscopic). (A)** The mass was a cystic lesion, 3.0 x 1.5 cm in size, filled with yellowish thick fluid. **(B)** The histopathology revealed a chronic granulomatous inflammation consisting of scattered epithelioid histiocytes, Langhans giant cells (*yellow arrow*) and areas of caseous necrosis, suggestive of a mycobacterial disease process (H&E stain, x100; inlet x400).

An 8-month-old Korean female infant presented with a rapidly growing erythematous subcutaneous nodule on the anterior chest wall noted 10 days previously. She had no history of trauma and a complete immunization history. There were no constitutional symptoms, such as fever, chilling, cough, sputum, sweating and weight loss. Seven months ago she was vaccinated in her 4^th^ week of life with intradermal BCG vaccine (Danish strain 1331, Statens Serum Institut, Denmark ) and the vaccination site on her left deltoid healed to leave a scar without any wound complications. A tuberculin skin test was not performed. The physical examination revealed a tender erythematous immobile firm mass along the left second intercostal space of the midclavicular line, measuring about 3 x 2 cm in size (Figure 1). There was no lymphadenopathy or palpable lymph node. The laboratory evaluation demonstrated a WBC count of 12.780 × 10^3^/μL (64% neutrophils, 17% lymphocytes and 19% monocytes), an elevated erythrocyte sedimentation rate (39 mm/hr) and C-reactive protein levels (1.74 mg/dL). Other laboratory investigations including immune study were within normal ranges. Chest X-ray and CT showed a 3 x 2 cm-sized oval subcutaneous mass with soft tissue density without any associated pulmonary parenchymal lesion. On the chest CT, the mass was ill-defined with abscess formation and infiltrated into the surrounding subcutaneous fat and pectoralis major muscle (Figure 2). Under the impression of a chest wall hot abscess or inflamed epidermal cyst, empirical antibiotics were started and an en bloc resection of the mass under general anesthesia was undertaken including the adjacent infiltrated tissue. The mass was a cystic lesion, 3.0 x 1.5 cm in size and filled with yellowish thick fluid. The histopathology revealed a chronic granulomatous inflammation consisting of scattered epithelioid histiocytes, Langhans’ type giant cells and areas of caseous necrosis, suggestive of a mycobacterial disease process (Figure 3). Ziehl Neelsen stain for acid-fast bacteria was negative (Figure 3) and routine cultures of the cystic fluid were all negative for microorganisms including tuberculosis and fungi. The tissue PCR study was also negative for tuberculosis. There was no contact with tuberculosis to the patient in her family or with neighbors. Based on all these findings, diagnosis was made as a BCG vaccine complication, demonstrating with subacute character. Antibiotics were changed to an anti-tuberculosis regimen with isoniazid 15 mg/kg/day lasting for 6 months and the patient was in a good healthy status without any complications until 1 year of age on follow-up.

## Discussion

Because there is still a high prevalence of tuberculosis in Korea, the BCG vaccination is administered to all healthy infants as early as after birth. The BCG vaccination complications are classified into five different types: (1) local disease, (2) regional disease, (3) distant disease, (4) disseminated disease, and (5) other BCG syndromes [2,3]. Among those complications, the most common type of complication induced by a BCG vaccination is a regional lymphadenitis and a subcutaneous abscess around the administration site. According to this classification, this case may be included in the distant disease type [2,3].

Even though a chest wall abscess induced by tuberculosis is very rare in an infant, a BCG vaccine complication or tuberculosis should be also considered in the differential diagnosis of a chest wall abscess in this age group [3]. The tissue culture in a BCG- induced lymphadenitis is known to be negative for tuberculosis 20 weeks after the BCG vaccination [4]. In the present case, tissue culture of the lesion was also negative several months after BCG vaccination. Meanwhile, in the present case, the chest CT showed a peripherally enhancing chest wall abscess in the subcutaneous layer infiltrating the pectoralis muscle, which is consistent with a caseous abscess induced by tuberculosis. Like in the present case, if a chest wall abscess presents without the isolation of any microorganism and histopathologic findings are shown consistent with a mycobacterial disease process, the possibility of a chest wall abscess induced by tuberculosis should be considered. Although tuberculosis was neither confirmed by culture nor PCR study, we concluded that the chest wall abscess was induced by a BCG vaccination possibly via an hematogenously spread mechanism, because of its histopathologic consistency with tuberculosis, no pulmonary parenchymal tuberculosis lesion, no isolation of microorganism including tuberculosis, the occurrence in a healthy infant and within 1 year of BCG vaccination and no contact to patients with tuberculosis. However, the exact pathogenesis remained unclear.

Granulomas in BCG diseases usually present as cold abscesses [4]. However, our case interestingly appeared as a hot abscess with overlying cutaneous erythema, which is thought to be preceded by a cold abscess. And the erythema might result from the direct extension of the existing abscess inflammation to the overlying skin or a secondary other bacterial infection in the overlying skin and/or the abscess lesion, which is likely supported by the laboratory findings on admission.

## Conclusion

In conclusion, although very rare, a BCG disease should be considered in the differential diagnosis of chest wall abscess, even if presenting as hot abscess, in infants, even immunocompetent, with an age related to BCG vaccination complications.

## Consent

Written informed consent was obtained from the patient for publication of this Case report and any accompanying images. A copy of the written consent is available for review by the Editor-in-Chief of this journal. This case study was approved by Institutional Review Board for Uijeongbu St. Mary’s Hospital (UC14ZISE0128).
